# Prevalence of Myopic Macular Features in Dutch Individuals of European Ancestry With High Myopia

**DOI:** 10.1001/jamaophthalmol.2021.5346

**Published:** 2021-12-16

**Authors:** Annechien E. G. Haarman, Milly S. Tedja, Corina Brussee, Clair A. Enthoven, Gwyneth A. van Rijn, Johannes R. Vingerling, Jan E. E. Keunen, Camiel J. F. Boon, Annette J. M. Geerards, Gré P. M. Luyten, Virginie J. M. Verhoeven, Caroline C. W. Klaver

**Affiliations:** 1 Erasmus Medical Center, Department of Ophthalmology, Rotterdam, the Netherlands; 2 Erasmus Medical Center, Department of Epidemiology, Rotterdam, the Netherlands; 3 Leiden University Medical Center, Department of Ophthalmology, Leiden, the Netherlands; 4 Radboudumc, Department of Ophthalmology, Nijmegen, the Netherlands; 5 Amsterdam University Medical Center, Department of Ophthalmology, Amsterdam, the Netherlands; 6 The Rotterdam Eye Hospital, Rotterdam, the Netherlands; 7 Erasmus Medical Center, Department of Clinical Genetics, Rotterdam, the Netherlands; 8 Institute of Molecular and Clinical Ophthalmology, Basel, Switzerland

## Abstract

**Importance:**

High myopia incidence and prevalence is increasing worldwide, and the visual burden caused by myopia is expected to rise accordingly. Studies investigating the occurrence of myopic complications in individuals of European ancestry with high myopia are scarce, hampering insights into the frequency of myopic retinal complications in European individuals and their visual burden.

**Objective:**

To assess the frequency of myopic macular features in individuals of European ancestry with high myopia.

**Design, Setting, and Participants:**

This cross-sectional analysis of the Dutch Myopia Study (MYST) and individuals with high myopia from the Rotterdam Study (RS) included 626 patients with high myopia (spherical equivalent of refractive error [SER] ≤−6 diopters [D] or axial length [AL] ≥26 mm) who underwent an extensive ophthalmic examination including multimodal retinal imaging. In addition to this combination of a population-based cohort study and mix-based high myopia study, a systematic literature review was also performed to compare findings with studies of individuals of Asian ancestry.

**Exposures:**

High myopia, age, and AL.

**Main Outcomes and Measures:**

Frequency of myopic macular and optic disc features: tessellated fundus, myopic macular degeneration (MMD), staphyloma, peripapillary intrachoroidal cavitation, peripapillary atrophy (PPA), and “plus” lesions (choroidal neovascularization, Fuchs spot, and lacquer cracks).

**Results:**

The mean (SD) SER of the combined study population (MYST and RS) was −9.9 (3.2) D; the mean (SD) age was 51.4 (15.1) years, and 387 (61.8%) were women. The prevalence of MMD was 25.9% and increased with older age (*P* for trend <.001), lower SER (odds ratio [OR], 0.70; 95% CI, 0.65-0.76; *P* < .001), and higher AL (OR, 2.53; 95% CI, 2.13-3.06; *P* < .001). Choroidal neovascularization or Fuchs spot was present in 2.7% (n = 17), both lesions in 0.3% (n = 2), and lacquer cracks in 1.4% (n = 9). Staphyloma, PPA, and MMD were highly prevalent in visual impaired and blind eyes (frequency was 73.9% [20 of 27], 90.5% [19 of 21], and 63.0% [17 of 27] of unilateral blind eyes for MMD, staphyloma, and PPA, respectively). Seven previous studies in Asian populations reported a variable MMD frequency ranging from 8.3% to 64%, but frequencies were similar for comparable risk profiles based on age and SER.

**Conclusions and Relevance:**

In this cross-sectional study of a highly myopic Dutch population of European ancestry, myopic retinal features were frequent; were associated with age, SER, and AL; and occurred in all visually severely impaired eyes. The absence of treatment options for most of these retinal complications emphasizes the need for effective strategies to prevent high myopia.

## Key Points

**Question:**

What is the prevalence of myopic macular degeneration in Dutch individuals of European ancestry with high myopia?

**Findings:**

In this cross-sectional study of 626 individuals with European ancestry with high myopia, the prevalence of myopic macular degeneration was 25.9% and increased with older age, lower spherical equivalent of refractive error, and higher axial length.

**Meaning:**

Myopic retinal features were frequent in this highly myopic study population, but not different than patients of Asian ancestry with similar risk profiles.

## Teaser

This cross-sectional study assesses the frequency of myopic macular features in individuals of European ancestry with high myopia.

## Introduction

Myopia is a refractive error predominantly caused by eye elongation, which can lead to irreversible loss of vision, including among those in the working-age population.^1^ The prevalence of myopia is increasing globally. It is estimated that in 2050, half of the world population will be myopic, and 10% will be highly myopic.^2^ As a result, the visual burden of myopia is expected to increase accordingly. One of the major causes of visual impairment (VI) owing to myopia is myopic macular degeneration (MMD), also termed *myopic maculopathy* or *myopic retinopathy*.^3^ According to recent estimations for 2050, VI owing to MMD will grow from 10.0 million to 55.7 million people worldwide (prevalence, 0.57%; 95% CI, 29.0 million to 119.7 million), and blindness owing to MMD will grow from 3.3 million to 18.5 million (prevalence, 0.19%; 95% CI, 9.6 million to 39.7 million).^4^

The definition of MMD is highly variable among studies but generally includes myopic retinal characteristics, such as tessellated fundus, lacquer cracks, myopic chorioretinal atrophy, and staphyloma.^5,6^ Pathologic myopia is another frequently used concept and includes posterior staphyloma, myopic choroidal neovascularization (CNV), Fuchs spot, lacquer cracks, or myopic maculopathy equal to or more serious than diffuse choroidal atrophy.^7^ Most of these features are directly related to axial length (AL) elongation and thinning of the retina, attenuation of the retinal pigment epithelium, and choroidal thinning.^5,8,9,10,11^ The variability in classifications hindered direct comparisons,^12,13,14^ but the Meta-analysis for Pathologic Myopia (META-PM) Study Group international grading system developed in 2015 changed the grading landscape by introducing a uniform classification for high myopia features.^5^ A potential limitation of this system was that its development was primarily based on clinical data of Asian patients. European studies using this classification system in individuals with high myopia are scarce,^15^ hampering insights into the frequency of myopic retinal complications in European individuals and their visual burden.

In this cross-sectional study, we describe the occurrence of myopic macular features in individuals with high myopia from the population-based Rotterdam Study (RS) and the Dutch Myopia Study (MYST). This knowledge will increase our understanding of the morbidity of high myopia and may guide prevention and treatment strategies.

## Methods

### Study Population

For the current study, we included persons with spherical equivalent of refractive error (SER) of −6 diopters (D) or less and AL of 26 mm or greater from the Dutch population-based RS (n = 117) and MYST (n = 509). The RS consists of 3 population-based cohorts of persons aged 45 years and older.^16,17^ MYST was conducted from 2010 to 2012; participants were recruited via public media, eye care clinicians, and announcements on websites from the study or from Erasmus Medical Center. Details of both studies can be found in the eMethods and eFigures 1 and 2 in the Supplement. Both studies adhered to the tenets of the Declaration of Helsinki and were approved by the local ethics committees of Erasmus Medical Center, Rotterdam, the Netherlands (MEC-02-1015 [RS] and MEC 2009-248 [MYST]). Written informed consent was obtained from all study participants.

### Ophthalmological Examination

Both studies included extensive ophthalmological examinations at the same research center using the same protocol. This included SER measurements with Topcon RM-A2000 Auto-Refractor (Topcon Optical Company) and AL measurements with Lenstar LS900 (Laméris Ootech), or with the A-scan function of PacScan 300 AP (Sonomed Escalon) for participants with AL of 30 mm or greater. After pharmacologic mydriasis with phenylephrine and tropicamide, we performed retinal imaging including stereoscopic 35° digital color fundus photography of the macula and optic disc (Topcon TRC-50EX; Topcon Optical Company, with a Sony DXC-950P digital camera, 0.44 megapixel; Sony Corporation); optical coherence tomography (OCT) (Topcon 1000, 2000 [SD] and Topcon-DRI [SS]; Topcon Optical Company); infrared imaging and autofluorescence (Heidelberg HRA-2, Heidelberg Engineering).

### Grading of Myopic Features

We graded the retinal images using information from all modalities (fundus photography, OCT, infrared imaging, and autofluorescence). We performed META-PM grading on the color fundus photographs^5^ and used the E3 OCT classification to grade the OCTs.^18^ Details of the grading can be found in the eMethods and eFigure 3 in the Supplement.

### Statistical Analysis

The SER was calculated by adding half the cylindrical value to the spherical value. Mean SER of both eyes was calculated; SER of only 1 eye was used when both eyes were not available. We selected the eye with the most severe myopic pathology according to the META-PM classification for analysis. For the analyses assessing VI and blindness, we evaluated both eyes. Descriptive statistics of MMD features were presented as age-specific frequencies. We performed logistic regression analyses, adjusted for age and sex, to assess the association between MMD lesions or META-PM categories and AL or SER; and adjusted for sex and SER to assess the association between MMD lesions and age. We performed a linear trend test to investigate the association between age and MMD or META-PM category. We investigated co-occurrence of lesions in eyes with CNV, staphyloma, and MMD using χ^2^ or Fisher exact test.^14^

Unilateral and bilateral VI was defined as a best-corrected visual acuity (BCVA, decimal [Snellen]) less than 0.3 (20/70) and 0.05 or greater (20/400) and blindness as BCVA less than 0.05 (20/400) according to the World Health Organization definition.^19^ We assessed the association between VI or blindness and AL using an analysis of variance test. The IBM SPSS Statistics, version 25 (IBM Corporation) was used for the statistical analyses. For all analyses, a 2-sided *P* < .05 was considered statistically significant. No adjustment for multiple analyses was performed.

### Comparison With Asian Populations: Systematic Review

To compare our findings in highly myopic persons with European ancestry with myopic pathology occurring in Asian individuals, we performed a systematic review of all Asian high myopia studies. A detailed description of the systematic review to identify studies among populations or patients with Asian ancestry can be found in the eMethods and eFigure 4 in the Supplement.

## Results

Participants of RS had a mean (SD) age of 69.2 (10.4) years, and 48 (41.0%) were men (Table 1). The mean (SD) SER was −8.4 (2.7) D, and the mean (SD) AL was 26.5 (1.2) mm. The mean (SD) age of the MYST study population was 47.3 (12.9) years, and 191 (37.5%) were men (Table 1). MYST participants had higher degrees of myopia (mean [SD] SER, −10.3 [3.3] D); 244 of 509 (47.9%) had a SER between −6 D and −10 D, 154 of 509 (30.3%) had a SER between −10 D and −15 D, and 38 of 509 (7.5%) had a SER of −15 D or less. Mean (SD) AL was 27.5 (1.7) mm.

**Table 1.** Baseline Characteristics of Rotterdam Study and Myopia Study (MYST) Participants With META-PM Classification Data

Characteristic	No. (%)		
	Rotterdam Study (n = 117)	MYST (n = 509)	Total (n = 626)
Age, y			
Mean (SD) [range]	69.2 (10.4) [47.5 to 91.9]	47.3 (12.9) [23.0 to 79.6]	51.4 (15.1) [23.0 to 91.9]
% Missing	0	0	0
Age categories, y			
20-29	0	65 (12.8)	65 (10.4)
30-39	0	94 (18.5)	94 (15.0)
40-49	4 (3.4)	120 (23.6)	124 (19.8)
50-59	21 (17.9)	142 (27.9)	163 (26.0)
60-69	36 (30.8)	72 (14.1)	108 (17.3)
70-79	40 (34.2)	16 (3.1)	56 (8.9)
>80	16 (13.7)	0	16 (2.6)
Sex^a^			
Female	69 (59.0)	318 (62.5)	387 (61.8)
Male	48 (41.0)	191 (37.5)	239 (38.2)
% Missing	0	0	0
SER			
No.	117	436	553
Mean (SD) [range], D	−8.4 (2.7) [−19.1 to −6.0]	−10.3 (3.3) [−23.9 to −6.0]	−9.9 (3.2) [−23.9 to −6.0]
% Missing	0	14.3	13.2
SER in different age categories, y, mean (SD) [No.], D			
20-29	NA	−9.6 (2.2) [62]	−9.6 (2.2) [62]
30-39	NA	−10.2 (2.9) [86]	−10.2 (2.9) [86]
40-49	−7.3 (1.0) [4]	−10.7 (3.8) [104]	−10.6 (3.8) [108]
50-59	−7.6 (1.6) [21]	−10.4 (3.3) [115]	−10.0 (3.3) [136]
60-69	−8.9 (2.9) [36]	−10.1 (3.3) [60]	−9.7 (3.2) [96]
70-79	−8.5 (3.0) [40]	−9.9 (3.8) [9]	−8.7 (3.2) [49]
>80	−8.7 (2.9) [16]	NA	−8.7 (2.9) [16]
SER worse eye			
No.	117	436	553
Mean (SD) [range], D	−8.9 (3.4) [−20.0 to −5.3]	−10.4 (3.3) [−25.38 to −5.13]	−10.1 (3.4) [−25.4 to −5.13]
% Missing	0	14.3	13.2
AL			
No.	65	507	571
Mean (SD) [range], mm	26.5 (1.2) [24.4 to 31.3]	27.5 (1.7) [24.2 to 37.6]	27.4 (1.6) [24.2 to 37.6]
% Missing	44.4	0.39	9.6
AL worse eye			
No.	65	507	572
Mean (SD) [range], mm	26.7 (1.5) [24.4 to 31.7]	27.5 (1.8) [24.0 to 37.4]	27.4 (1.8) [24.0 to 37.4]
% Missing	44.4	0.4	9.4

Abbreviations: AL, axial length; D, diopters; META-PM, Meta-analysis for Pathologic Myopia; NA, not applicable; SER, spherical equivalent of refractive error.

^a^ Sex was determined by the investigator.

### Validation for Pooled Analysis

To maximize the study size of individuals with high myopia, we favored a pooled analysis of both study populations. Because the sampling strategy of MYST may jeopardize representation of a general population, we first investigated potential selection bias by comparing the frequency of MMD in both studies. In a crude analysis, we observed a higher frequency of MMD in RS (MYST vs RS, 24.0% vs 34.2%; *P* = .02). Because RS included more participants of older age, we also calculated the risk of MMD in MYST compared with RS adjusted for age and sex (risk of MMD in MYST in reference to RS: odds ratio [OR], 1.02; 95% CI, 0.51-2.06), suggesting that selection for severe myopic pathology was unlikely in MYST. We therefore decided that findings from a pooled analysis reliably represented population frequencies. The demographic characteristics of the combined study population (n = 626) are summarized in Table 1.

### Prevalence of Myopic Retinal Features

**Figure 1.**
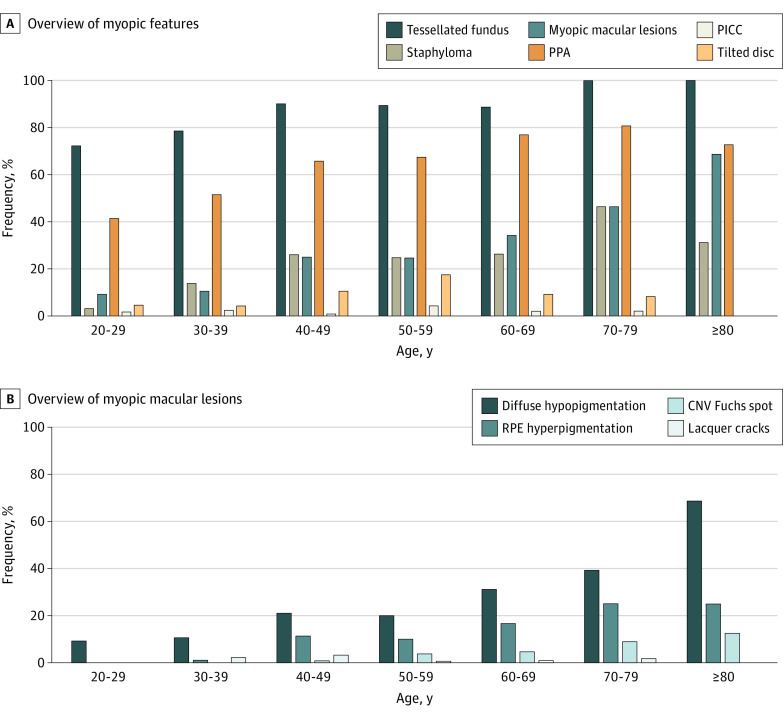
Frequency of Various Myopic Features per Age Category A, Complete overview of myopic features; B, Detailed overview of the myopic macular lesions. CNV indicates choroidal neovascularization; PICC, peripapillary intrachoroidal cavitation; RPE, retinal pigment epithelium; PPA, peripapillary atrophy.

**Figure 2.**
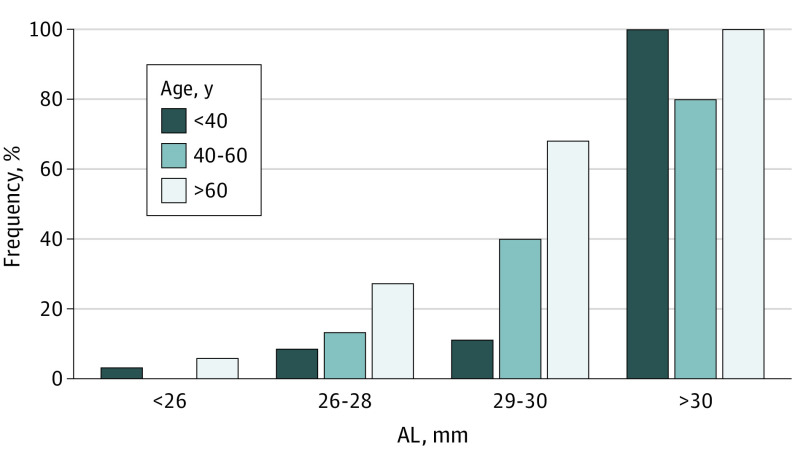
Association Between the Frequency of Myopic Macular Degeneration and Axial Length (AL) and Age

Myopic retinal features in individuals with high myopia as a function of age can be found in Figure 1. Tessellated fundus and peripapillary atrophy (PPA) were highly frequent in all age categories (47 of 65 [72.3%] and 27 of 65 [41.5%], respectively, in patients aged 20-29 years, to 100% and 80.8% in patients aged 70-79 years). Staphylomas and retinal pigment epithelium hyperpigmentation were scarce in those younger than 30 years but rose strongly in frequency with age up to 43.0% and 25%, respectively, in those 70 years and older. Risks of most lesions were associated with age, AL, and SER (eTable 1 in the Supplement); Figure 2 and eFigure 5 in the Supplement show the association between age and AL for MMD, tessellated fundus, and staphyloma. In eyes with AL greater than 30 mm, the frequency of most lesions was high but did not show a linear association with increasing age. The occurrence of peripapillary intrachoroidal cavitation was rare (n = 13 [2.4%]); the highest frequency was observed in patients aged 50-60 years (n = 6 [4.3%]). The highest frequency of tilted disc was also observed in this age category (n = 28 [17.6%]). The occurrence of retinoschisis was rare (n = 1). This patient also presented with tessellated fundus and staphyloma. Lamellar hole also occurred in only 1 patient with high myopia (SER, −10.8 D).

### Prevalence of MMD

Myopic macular degeneration included all myopic macular pathology and excluded tessellated fundus. The frequency of MMD in the combined cohort was 25.9% and increased with age (*P* for trend <.001), lower SER (OR, 0.70; 95% CI, 0.65-0.76), and higher AL (OR, 2.53; 95% CI, 2.13-3.06) (Table 2; eTables 2 and 3 in the Supplement). In younger patients, however, MMD was not rare: the MMD frequency was 9.2% (6 of 65) in individuals with high myopia aged 20 to 29 years, 10.6% (10 of 94) in those aged 30 to 39 years, and 25.0% (31 of 124) in patients aged 40 to 49 years. Diffuse chorioretinal atrophy (META-PM category C2) was the most prevalent MMD feature (115 [18.4%]), followed by myopic macular atrophy (C4, 30 [4.8%]) and patchy chorioretinal atrophy (C3, 15 [2.4%]). Foveal sparing of chorioretinal atrophy was present in 30 of 68 (44.1%). The occurrence of META-PM “plus” lesions (ie, CNV, Fuchs spot, or lacquer cracks) was rare: CNV or Fuchs spot was present in 2.7% (n = 17), presence of both lesions was present in 0.3% (n = 2), and presence of lacquer cracks was seen in 1.4% (n = 9). Patients with older age were more frequently affected by more severe MMD categories, ie, MMD with macular (C4) or patchy chorioretinal atrophy (C3) (eFigure 6 in the Supplement).

**Table 2.** Frequency of Myopic Macular Degeneration (MMD) and META-PM Categories 2-4 in the Combined Cohort of the Rotterdam Study and Myopia Study (n = 626)^a^

Factor	Total, No.	MMD (n = 162)	META-PM category 2: diffuse chorioretinal atrophy (n = 115)	META-PM category 3: patchy chorioretinal atrophy (n = 15)	META-PM category 4: myopic macular atrophy (n = 30)
Total	626	162 (25.9)	115 (18.4)	15 (2.4)	30 (4.8)
Age, mean (95% CI) [No.], y		58.6 (56.3-60.8) [162]	58.2 (55.3-61.0) [115]	58.2 (52.9-63.6) [15]	59.7 (54.9-64.5) [30]
Age group, y					
20-29	65	6 (9.2)	6 (9.2)	0	0
30-39	94	10 (10.6)	10 (10.6)	0	0
40-49	124	31 (25.0)	19 (15.3)	4 (3.2)	8 (6.5)
50-59	163	40 (24.5)	27 (16.6)	4 (2.5)	8 (4.9)
60-69	108	38 (35.2)	24 (22.2)	6 (5.6)	8 (7.4)
70-79	56	26 (46.4)	20 (35.7)	1 (1.8)	4 (7.1)
>80	16	11 (68.8)	9 (56.3)	0	2 (12.5)
*P* value for trend		<.001	<.001	.10	.002
Male	239	62 (25.9)	40 (16.7)	5 (2.1)	16 (6.7)
Female	387	100 (25.8)	75 (19.4)	10 (2.6)	14 (3.6)
Comparison sex^b^		.99	.41	.70	.08
SER, mean (95% CI) [No.], D	553	−12.7 (−13.4 to −12.0) [140]	−12.1 (−12.9 to −11.4) [104]	−14.3 (−17.3 to −11.3) [11]	−14.4 (−16.5 to −12.3) [24]
OR (95% CI)^c^		0.70 (0.65 to 0.76)	0.81 (0.76 to 0.86)	0.79 (0.69 to 0.89)	0.76 (0.69 to 0.84)
Axial length, mean (95% CI) [No.], mm	572	29.2 (28.8 to 29.5) [137]	28.7 (28.4 to 29.1) [94]	29.6 (28.2 to 31.0) [13]	30.5 (29.5 to 31.5) [28]
OR (95% CI)^c^		2.55 (2.13 to 3.06)	1.56 (1.37 to 1.77)	1.58 (1.26 to 1.97)	2.02 (1.65 to 2.48)
BCVA, mean (95% CI) [No.], Snellen (decimal)		0.59 (0.53 to 0.65) [158]	0.70 (0.64 to 0.76) [111]	0.55 (0.33 to 0.77) [15]	0.23 (0.13 to 0.34) [30]
Approximate Snellen equivalent at 20 ft		20/33	20/28	20/36	20/87
Comparison BCVA^d^		<.001	0.70 [Reference]	0.13	<.001

Abbreviations: BCVA, best-corrected visual acuity; D, diopters; OR, odds ratio; SER, spherical equivalent of refractive error.

^a^ Frequencies are stratified by age and sex. Mean SER, axial length, and BCVA are also shown. MMD according to the Meta-analysis for Pathologic Myopia (META-PM) classification system was defined as META-PM category 2 or higher or presence of any “plus” lesions (ie, choroidal neovascularization, Fuchs spot, lacquer cracks). Sex was determined by the investigator.

^b^ *P* value χ^2^ test.

^c^ Adjusted for age and sex.

^d^ *P* value independent-samples *t* test.

### Visual Consequences of MMD

**Figure 3.**
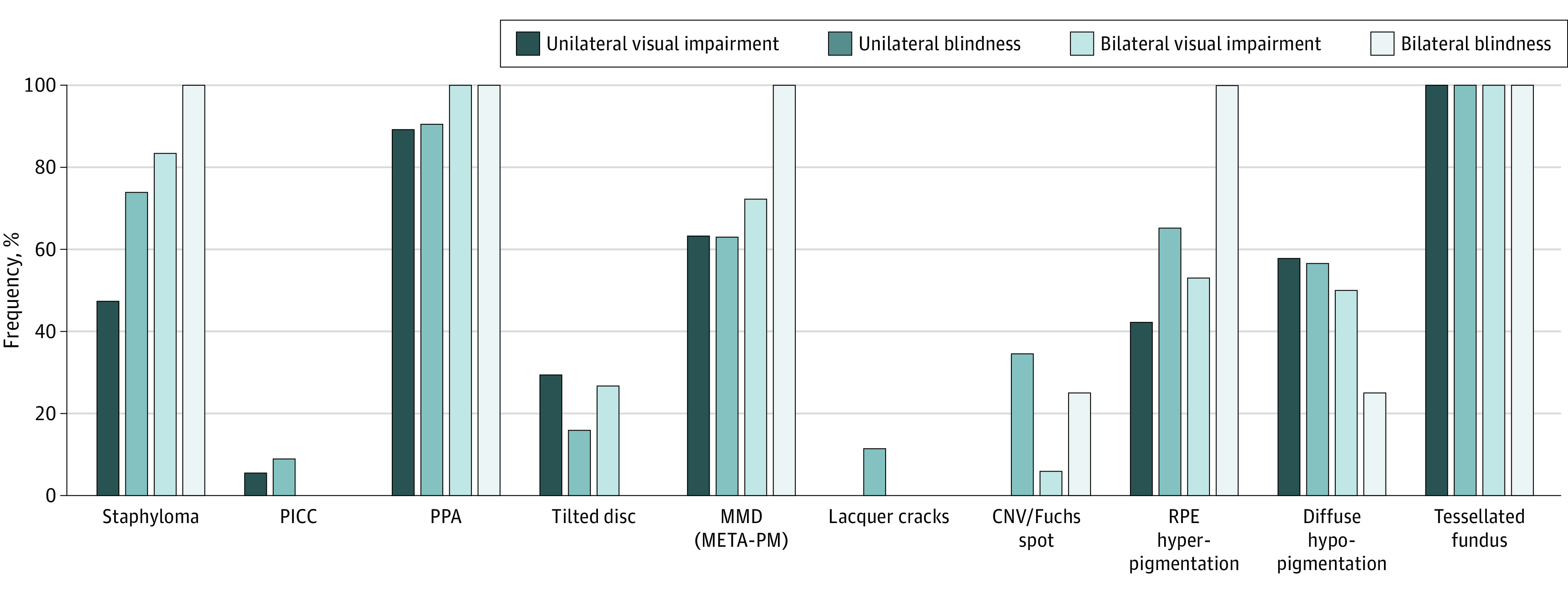
Frequency of Various Retinal Myopic Features in the Affected Eyes of Patients With Visual Impairment or Blindness CNV indicates choroidal neovascularization; META-PM, Meta-analysis for Pathologic Myopia; MMD, myopic macular degeneration; PICC, peripapillary intrachoroidal cavitation; PPA, peripapillary atrophy; RPE, retinal pigment epithelium.

Eyes with myopic macular atrophy (C4) had a lower visual acuity than diffuse or patchy chorioretinal atrophy. Mean (approximate Snellen equivalent) BCVA was 0.23 (20/87) (95% CI, 0.13-0.34), 0.55 (20/36) (95% CI, 0.33-0.77), and 0.70 (20/29) (95% CI, 0.64-0.76), respectively, for macular atrophy (C4), patchy chorioretinal atrophy (C3), and diffuse chorioretinal atrophy (C2) (*P* = .13 and *P* < .001 for C2 vs C3 and C2 vs C4, respectively) (Table 2). Unilateral VI occurred in 19 (3.1%) participants, unilateral blindness occurred in 28 (4.5%), and bilateral VI and blindness occurred in 10 (1.6%) and 2 (0.3%), respectively. In participants younger than 40 years, bilateral and unilateral VI or blindness occurred less often (n = 1 [0.2%]) than in older patients (n = 58 [9.3%]; *P* < .001) and occurred only in eyes with AL of 32 mm or greater. The risk of blindness increased with older age and longer AL. In 1 person, the unilateral blindness was owing to traumatic injury, for which he received an artificial eye; the myopic features in the remaining visually impaired participants (n = 70 eyes) are presented in Figure 3. Myopic macular degeneration was present in all bilaterally blind eyes, in 17 of 27 (63.0%) eyes with unilateral blindness, 13 of 18 (72.2%) with bilateral VI, and 12 of 19 (63.2%) with unilateral VI. Staphyloma and PPA were also highly frequent in visually impaired eyes (73.9% [20 of 27] and 90.5% [19 of 21] in unilateral blind eyes). Best-corrected visual acuity was worse in eyes with lesions closer to the fovea (more central in the Early Treatment Diabetic Retinopathy Study grid). Although a confluent patch of atrophy seemed to have a worse visual outcome compared with isolated patches, after correction for age, sex, pattern, and size, only the location of a lesion was associated with visual acuity, meaning that visual acuity was better in eyes with lesions located further away from the fovea (increase in BCVA was 0.133 [20/150] per disc area further away from the fovea; *P* = .007).

### Co-occurrence of Lesions

Lesions often occurred in the same eye. We observed that in eyes with CNV/Fuchs spot, the frequency of lacquer cracks (5.6% vs 1.3%; *P* = .23), diffuse hypopigmentation (72.2% vs 21%; *P* < .001), and MMD (100% vs 23.6%; *P* < .001) was higher than in eyes without a CNV/Fuchs spot (eFigure 7 in the Supplement).^14^ The frequency of tessellated fundus, hypopigmentation, lacquer cracks, and MMD was also higher in eyes with staphyloma (without staphyloma vs with staphyloma, 83.6% vs 99.3%; 12.0% vs 56.6%; 0% vs 6.2%; and 13.5% vs 64.4%, respectively, all *P* < .001).

### Comparison With Asian Populations With High Myopia

To assess the global perspective of our European data, we compared our frequencies with 10 studies investigating MMD in Asian individuals with high myopia: 2 conducted in Singapore, 1 in Taiwan, 1 in South Korea, 1 in Hong Kong, and 5 in China (eTable 4 in the Supplement).^20,21,22,23,24,25,26,27^ Wong et al^27^ described the occurrence of MMD in 3 different high myopia studies. The mean age of participants differed between these studies: 2 studies included mainly young patients (mean age, 20 years)^22,23^; 1 study included only older patients (≥65 years).^26^ The META-PM classification system was used in 8 of the 10 studies.^20,22,23,24,25^ Myopic macular degeneration was present in 8.3% to 72.7% compared with 25.9% in our combined study population (eTable 4 in the Supplement). In 1 study with a mean age comparable to the present study, MMD prevalence was 72.7%.^26^ Tessellated fundus was highly frequent in all studies except for the hospital-based study by Chen et al^20^ (9.3%). This study had the highest frequency of myopic CNV (20.7%) compared with the other studies (3.53 to 0.9%). Diffuse chorioretinal atrophy, patchy chorioretinal atrophy, and myopic macular atrophy were most prevalent in the hospital-based study by Zhao et al^25^ (28.5%, 19.1%, and 7.0%, respectively). These features ranged from 4.6% to 20.6%, 1.9% to 5.8%, and 0.2% to 5.5%, respectively, in the other 5 studies.^20,21,22,23,24^ “Plus” lesions were on average more prevalent in the Asian studies compared with the present study (Asian [range] vs our study [mean], CNV: 0.2%-20.0% vs 1.1%; Fuchs’ spot: 0%-9.4% vs 1.6%; and lacquer cracks: 1%-29.1% vs 1.4%).

## Discussion

In our study among patients of European ancestry with high myopia, we diagnosed the retinal features tessellated fundus, staphyloma, PPA, and MMD at a high frequency on color fundus photographs. Age and AL were associated with presence of these lesions. Within every age category, we observed an increasing lesion frequency with increasing AL, and this became more profound with age. Tessellated fundus was already present in 70% of young adults and is rather a hallmark of high myopia and not a complication per se.^28^ Regarding MMD, this lesion was the least frequent in young adults (<40 years) but nevertheless affected a considerable proportion of patients (10% in individuals aged 20-40 years). In the young individuals with high myopia, severe VI or blindness owing to any myopic macular feature only appeared in eyes longer than 32 mm. The frequency of MMD was 51% in individuals 70 years and older with high myopia, and VI or blindness occurred in 16%. In comparison to reports in Asian individuals, the frequency of myopic macular pathology in European individuals appears to be comparable, in particular when taking AL and age into consideration.

### Strengths and Limitations

The results of this study should be viewed in light of their merits and limitations. To our knowledge, this is the first European high myopia study investigating myopic macular features according to the META-PM classification system at such a large scale. The use of this internationally acknowledged system enables comparison of frequencies with other ethnicity populations. Other strong points are the population-based catchment area of the study and stratification of features according to age and AL. The omission of these important characteristics in most other studies impedes a proper interpretation of the data. Among the limitations are the lack of data on prevalence of other retinal complications or features that require a high-resolution OCT for diagnosis. In addition, the current study did not use wide field retinal imaging. Consequently, we could not observe more peripherally located lesions or an equatorial staphyloma. Lastly, the cross-sectional design hinders determination of the order of retinal events. To overcome this limitation, we looked at co-occurrence of CNV and other retinal lesions.^14^ We observed that in particular, the frequency of lacquer cracks was higher in eyes with CNV/Fuchs spot compared with eyes without this lesion, suggesting that lacquer cracks precede CNV. The current META-PM classification is only based on fundus photographs; incorporation of all image modalities for classification of myopic features will improve across study comparisons.

Prevalence of MMD in the present study was 25.9%, which is higher than the prevalence of 8.6% (95% CI, 6.1%-11.9%) reported in individuals with high myopia of the European Gutenberg Health Study (GHS).^29^ This study population was slightly less myopic, with a mean SER of −7.38 D in the GHS vs −9.9 D in the present study, which may explain this difference. One US study using a less detailed classification system observed a high frequency of a wide range of posterior myopic pathology in Asian participants compared with White individuals in the US (43% vs 31%).^30^ Prevalence of MMD in Asian studies appeared to be highly variable (8%-72.7%). Several factors currently hamper valid comparison between ethnicity groups. An important drawback is the large difference in age and SER. For example, Koh et al^22^ included young male participants (mean age, 21 years) and found a low MMD prevalence of 8.3%, while Chen et al^20^ included patients with a mean age of 40.6 years and observed a prevalence of 64%. Other limitations are differences in study design, eg, the purely hospital-based setting of the Asian studies^22,23^ vs the more population-based sampling in our study. Nevertheless, both European and Asian studies found robust trends of higher prevalence rates with higher SER and AL.^13,26,31,32,33^

What genes are responsible for these myopic macular features? Studies investigating the genetic background specific for MMD are scarce, mainly limited to individuals of Asian ancestry, and mostly lack grading according to the META-PM classification.^34,35,36,37,38,39^ Only 1 genome-wide association study did so and identified a significant hit annotated to the *CCDC102B* gene for MMD stage 2 to 4.^36^ In contrast, a recent candidate gene study involving individuals of White and Asian ancestry using META-PM did not find any genetic variants for MMD.^38,39^ In our view, MMD is associated with age and AL, and gene-finding studies specifically focusing on MMD are likely to fail.

“Plus” lesions, ie, CNV, Fuchs spot, and lacquer cracks, were rare in our study population, which is in line with reports from the GHS. In the GHS, prevalence of CNV/Fuchs spot was 0.6%; lacquer cracks, 2.5%.^29^ A French hospital-based cross-sectional study reported an extremely low prevalence of 0.07% (95% CI, 0.03%-0.13%) for all “plus” lesions in individuals with myopia of −6 D.^40^ The frequency of lacquer cracks was slightly higher in Asian studies^20,21,22,23,24,25,26,27^ compared with MYST (1%-29%). Although the mean age of participants in the Asian study^20^ reporting a frequency of 29% was comparable to our population, this population was more myopic (mean [SD] SER, −11.40 [4.80] D). Lacquer cracks may also be reported more frequently in Asian individuals because of the more pigmented retina-choroid complex, facilitating diagnosis. Detection of lacquer cracks is clinically important because they are associated with high risk of developing CNV^41^ and therefore need close clinical monitoring.^42^

## Conclusions

In this cross-sectional study of individuals of European ancestry with high myopia, the prevalence of MMD was associated with AL, SER, and age, and not so much on ancestry. Because MMD is a major cause of VI and blindness and (high) myopia incidence is increasing globally,^2^ we expect the visual burden of MMD to rise accordingly. To save quality of life and productivity, future research needs to focus on development of innovative interventions to prevent these complications, and eye care clinicians should encourage myopia control.^43,44,45,46^

## Notes

Supplement.
eMethods.
**eFigure 1.** Selection process of study participants of the Rotterdam Study (RSI, II and III). **eFigure 2.** Selection process of study participants of the MYST study. **eFigure 3.** Examples of myopic features in European eyes on color fundus imaging. **eFigure 4.** Flow chart of systematic literature search investigating the occurrence of myopic macular degeneration in Asian high myopia studies. **eFigure 5.** Association between the frequency of tessellated fundus and axial length and age and staphyloma and axial length and age. **eFigure 6.** Frequency of all META-PM categories in different age categories. **eFigure 7.** Frequency of tessellated fundus, lacquer cracks, diffuse hypopigmentation and MMD in eyes with and without CNV or Fuchs’ Spot. **eTable 1.** Association between various myopic features and axial length, spherical equivalent and age. **eTable 2.** Frequency of Myopic Macular Degeneration (MMD) and Meta-PM Categories 2-4 in the Rotterdam Study. **eTable 3.** Frequency of Myopic Macular Degeneration (MMD) and Meta-PM Categories 2-4 in the MYST Study. **eTable 4.** Prevalence of various myopic complications concerning populations with Asian ethnicity.
