# Supplementary material for: Prevalence of Myopic Macular Features in Dutch Individuals of European Ancestry With High Myopia
Source: JAMA Ophthalmol. 2021 Dec 16;140(2):115–23. doi: 10.1001/jamaophthalmol.2021.5346 (PMC8678902; doi:10.1001/jamaophthalmol.2021.5346)
Supplement: Supplement. — eMethods. eFigure 1. Selection process of study participants of the Rotterdam Study (RSI, II and III). eFigure 2. Selection process of study participants of the MYST study. eFigure 3. Examples of myopic features in European eyes on color fundus imaging. eFigure 4. Flow chart of systematic literature search investigating the occurrence of myopic macular degeneration in Asian high myopia studies. eFigure 5. Association between the frequency of tessellated fundus and axial length and age and staphyloma and axial length and age. eFigure 6. Frequency of all META-PM categories in different age categories. eFigure 7. Frequency of tessellated fundus, lacquer cracks, diffuse hypopigmentation and MMD in eyes with and without CNV or Fuchs’ Spot. eTable 1. Association between various myopic features and axial length, spherical equivalent and age. eTable 2. Frequency of Myopic Macular Degeneration (MMD) and Meta-PM Categories 2-4 in the Rotterdam Study. eTable 3. Frequency of Myopic Macular Degeneration (MMD) and Meta-PM Categories 2-4 in the MYST Study. eTable 4. Prevalence of various myopic complications concerning populations with Asian ethnicity. [file jamaophthalmol-e215346-s001.pdf]

Supplemental Online Content

Haarman AEG, Tedja MS, Brussee C, et al. Prevalence of myopic macular features in Dutch individuals of European ancestry with high myopia. *JAMA Ophthalmol*. Published online December 16, 2021. doi:10.1001/jamaophthalmol.2021.5346

eMethods.

eFigure 1. Selection process of study participants of the Rotterdam Study (RSI, II and III).

eFigure 2. Selection process of study participants of the MYST study.

eFigure 3. Examples of myopic features in European eyes on color fundus imaging.

eFigure 4. Flow chart of systematic literature search investigating the occurrence of myopic macular degeneration in Asian high myopia studies.

eFigure 5. Association between the frequency of tessellated fundus and axial length and age and staphyloma and axial length and age.

eFigure 6. Frequency of all META-PM categories in different age categories.

eFigure 7. Frequency of tessellated fundus, lacquer cracks, diffuse hypopigmentation and MMD in eyes with and without CNV or Fuchs' Spot.

eTable 1. Association between various myopic features and axial length, spherical equivalent and age.

eTable 2. Frequency of Myopic Macular Degeneration (MMD) and Meta-PM Categories 2-4 in the Rotterdam Study.

eTable 3. Frequency of Myopic Macular Degeneration (MMD) and Meta-PM Categories 2-4 in the MYST Study.

eTable 4. Prevalence of various myopic complications concerning populations with Asian ethnicity.

This supplemental material has been provided by the authors to give readers additional information about their work.

© 2021 Haarman AEG et al. *JAMA Ophthalmology.*

eMethods

Study populations

The Rotterdam Study

The Rotterdam Study (RS) is a population-based cohort study in a well-defined district of Rotterdam, The Netherlands aimed to unravel the etiology, preclinical course, natural history and potential targets for intervention for chronic diseases in mid-life and late-life.[14,15] The participants were all extensively examined at baseline and at subsequent follow-up visits that took place every 5 years. All persons who entered the current analyses had either a mean spherical equivalent of refractive error (SER) ≤-6 diopters (D) or axial length (AL) ≥26 mm in case of bilateral pseudophakia, aphakia or refractive surgical procedures without data on refractive error before surgery (eFigure 1).

MYST

For the current study, we included 509 18+ year old highly myopic individuals from The MYopia STudy (MYST), a high myopia case control study which was executed in 2010-2012.[1] Participants were recruited for MYST via public media, eye care providers and announcements on the study website and Erasmus MC website. Persons with syndromic high myopia were excluded. All persons who entered the current analyses had either a mean SER ≤-6 D or AL ≥26 mm in case of bilateral pseudophakia, aphakia or refractive surgical procedures without data on refractive error before surgery (eFigure 2).

Grading system

The META-PM grading included tessellated fundus, diffuse hypopigmentation, RPE hyperpigmentation, myopic chorioretinal atrophy, choroidal neovascularization (CNV), Fuchs' Spot, lacquer cracks, staphyloma, peripapillary atrophy (PPA) and peripapillary intrachoroidal cavitation (PICC) (Figure 2).[5] Staphylomas were graded for presence, location and foveal involvement. PICC was

© 2021 Haarman AEG et al. *JAMA Ophthalmology.*

defined as an elevated, well-circumscribed, dome-shaped, yellow-orange lesion inferior to the optic disc along the inferior margin of the PPA.[18] The ovalness of the disc was determined to assess the optic disc tilting by calculating the ratio of the maximum diameter to the minimum diameter (the tilt ratio). A tilt ratio of more than 1.5 was defined as a tilted disc.[5] We used our other image modalities infrared and autofluorescence to verify lesions if needed. Inter-grader reliability of myopic lesions was assessed with additional grading of 25 randomly selected eyes by three graders (A.H., M.T, and C.B.); kappa statistics ranged from 0.67 [AH, MT] to 0.78 [CB, MT] and 0.86 [CB, AH]). In case of disagreement, consensus was reached during an expert [AH, MT, CB, CK]. The location of a lesion was determined within subfields of the ETDRS grid.[19] This means that when an edge of a lesion was located in the central ETDRS grid circle, it is marked as located in the central circle. Closer to the fovea means located more central in the ETDRS grid. Lesion size was determined as optic disc area (DA) and was categorized into four categories (<1DA; 1DA-<5DA; 5-<9DA and ≥9DA). Lesions were graded as isolated or confluent patches. Staging of pathology was in accordance with META-PM: category 0 was defined as absence of any retinal complication; category 1 as presence of a tessellated fundus; category 2 as presence of diffuse hypopigmentation, i.e. diffuse chorioretinal atrophy; category 3 as presence of one or more patches of myopic chorioretinal atrophy not located in the central circle; and category 4 as presence of myopic chorioretinal atrophy located in the central circle.[5] MMD was considered present in META-PM category ≥2 or presence of any ‘plus’ lesions (i.e. choroidal neovascularization (CNV), Fuchs’ Spot, lacquer cracks).[20]

Systematic review

We performed a systematic review in the PubMed database using the following MeSH terms: "Macular Degeneration/epidemiology", "Retinal Diseases/epidemiology”, "Aged”, "Middle Aged", "Adult", "Young Adult", "Adolescent", "Age Factors", "Cohort Studies", "Prevalence", "Incidence", "Case-Control Studies", "Cross-Sectional Studies", "Myopia", "Myopia, Degenerative" which resulted in 112 articles (eFigure 3). Titles and abstracts of articles were reviewed for relevancy and included

© 2021 Haarman AEG et al. *JAMA Ophthalmology.*

when the following criteria were met: (a) full text available, (b) written in English, (c) subject of article was myopic macular degeneration. We excluded studies on peripheral retinal lesions such as retinal detachment. A manual search was additionally performed by screening references of the included articles. Observational studies including high myopes graded according to META-PM or Avila were considered for inclusion in this study (N=7).

© 2021 Haarman AEG et al. *JAMA Ophthalmology.*

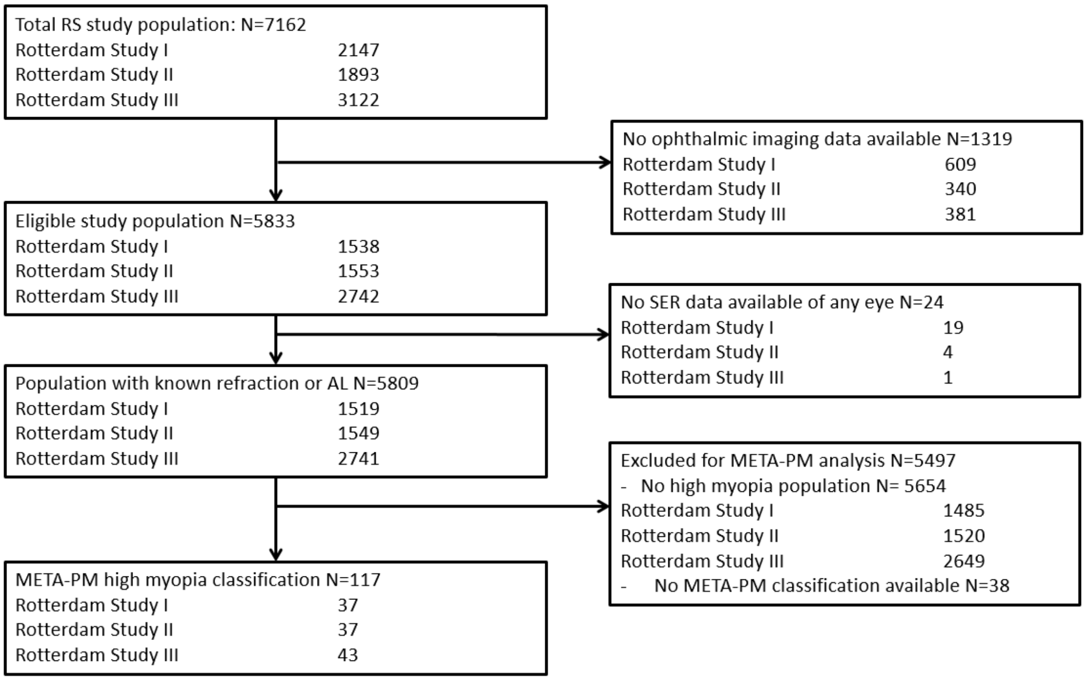

eFigure 1. Selection process of study participants of the Rotterdam Study (RSI, II and III).
Abbreviations: RS=Rotterdam Study; SER= spherical equivalent; AL= axial length.

© 2021 Haarman AEG et al. *JAMA Ophthalmology.*

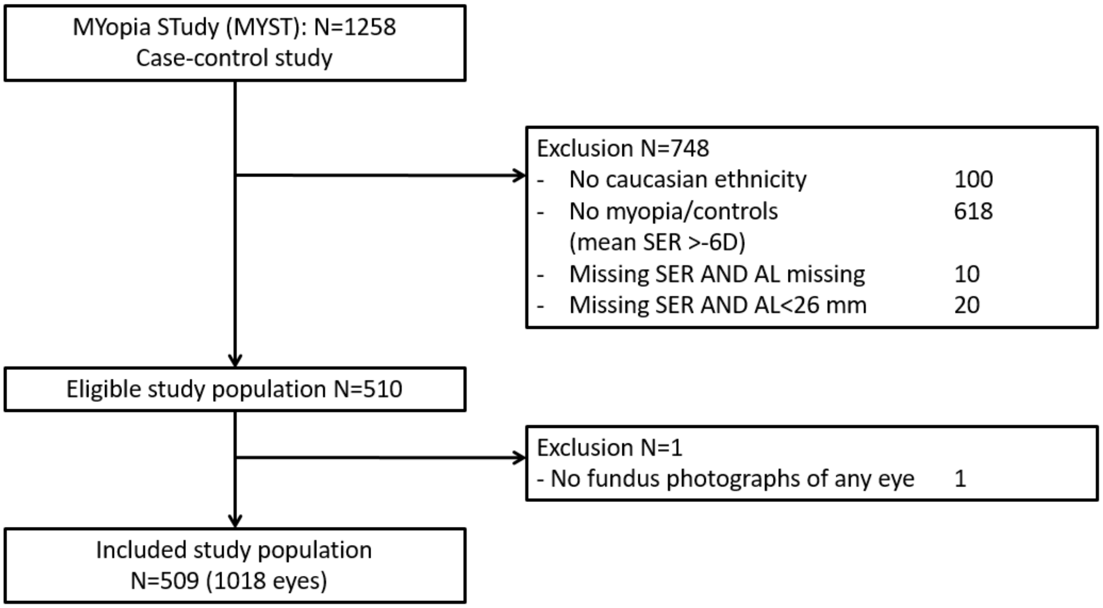

eFigure 2. Selection process of study participants of the MYST study. *Abbreviations: MYST=Myopia Study; SER= spherical equivalent; AL= axial length.*

© 2021 Haarman AEG et al. *JAMA Ophthalmology.*

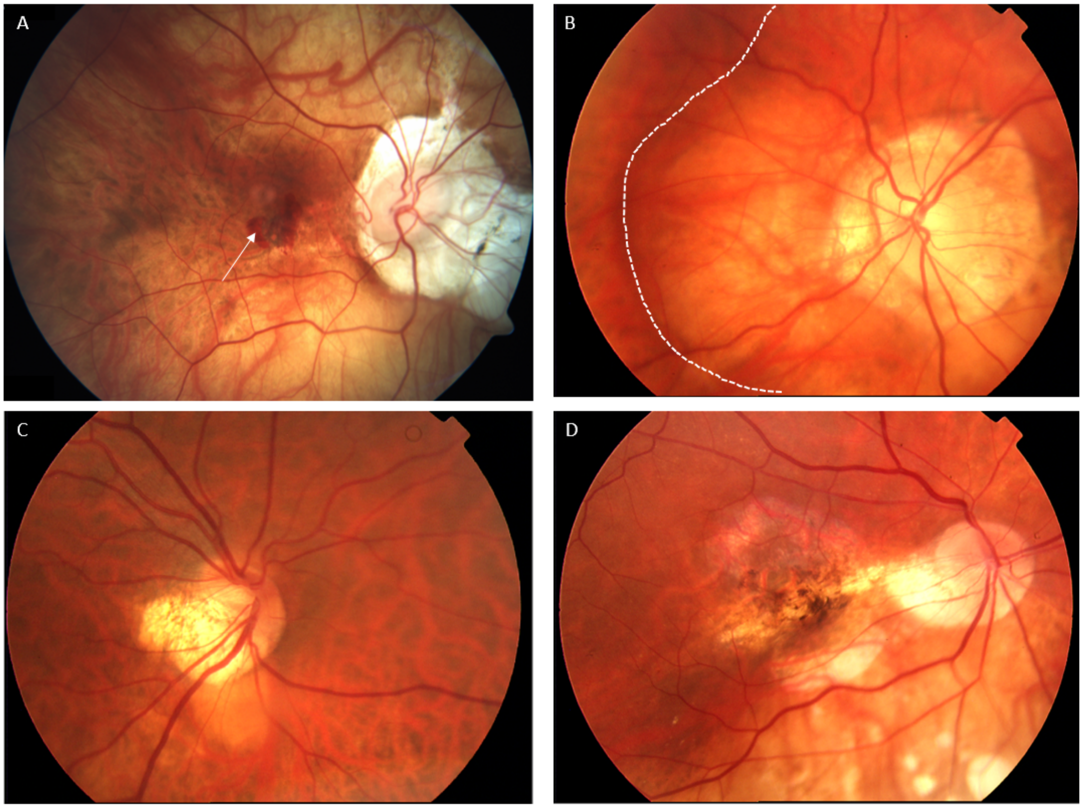

eFigure 3. Examples of myopic features in European eyes on color fundus imaging (A-D). *A: CNV, diffuse hypopigmentation and PPA; B: staphyloma with diffuse hypopigmentation, tilted disc and PPA; C: tessellated fundus, PPA and PICC; D: Fuchs' spot, patchy chorioretinal atrophy, diffuse hypopigmentation and PPA. White arrow in panel A indicates CNV. white dashed line in panel B indicates staphyloma. Abbreviations: CNV= choroidal neovascularization; RPE= retinal pigment epithelium; PPA = peripapillary atrophy; PICC = peripapillary intrachoroidal cavitation.*

© 2021 Haarman AEG et al. *JAMA Ophthalmology.*

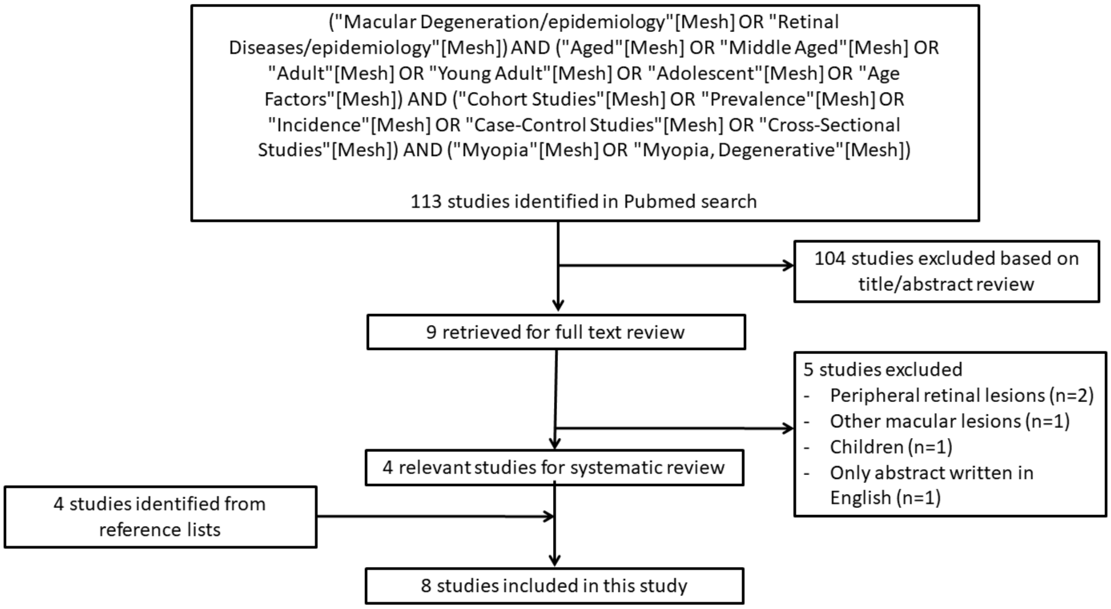

eFigure 4. Flow chart of systematic literature search investigating the occurrence of myopic macular degeneration in Asian high myopia studies.

© 2021 Haarman AEG et al. *JAMA Ophthalmology.*

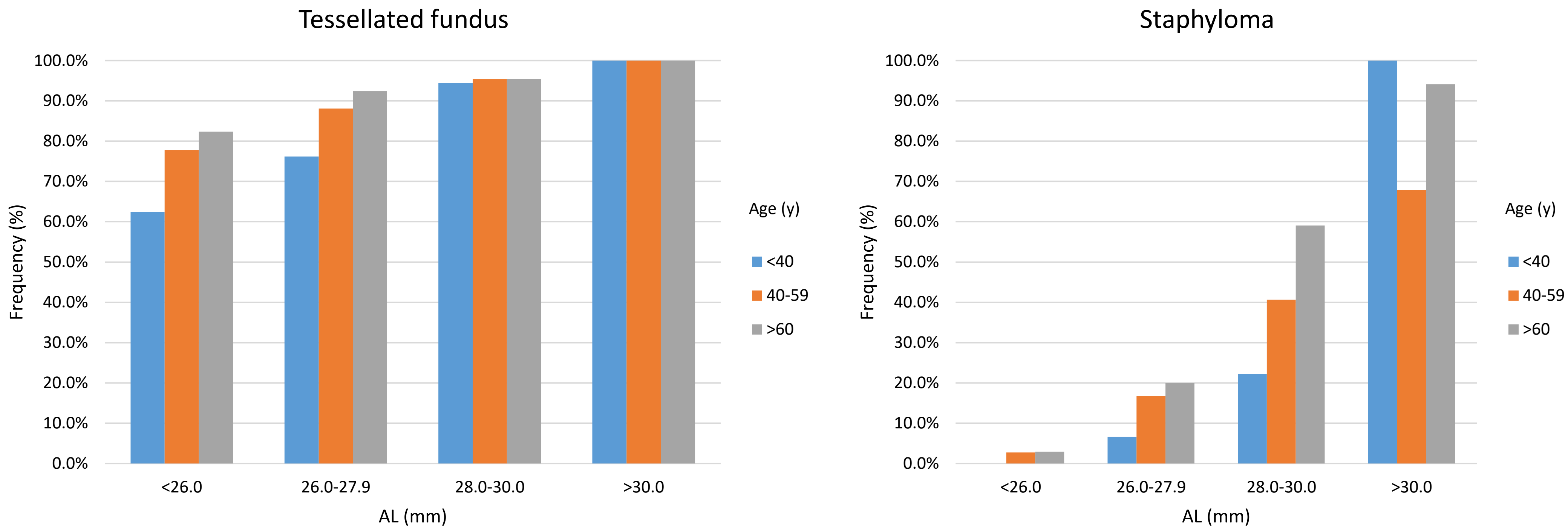

eFigure 5. Association between the frequency of tessellated fundus and axial length and age (left panel) and staphyloma and axial length and age (right panel). *Abbreviations: AL = axial length; y=years.*

© 2021 Haarman AEG et al. *JAMA Ophthalmology.*

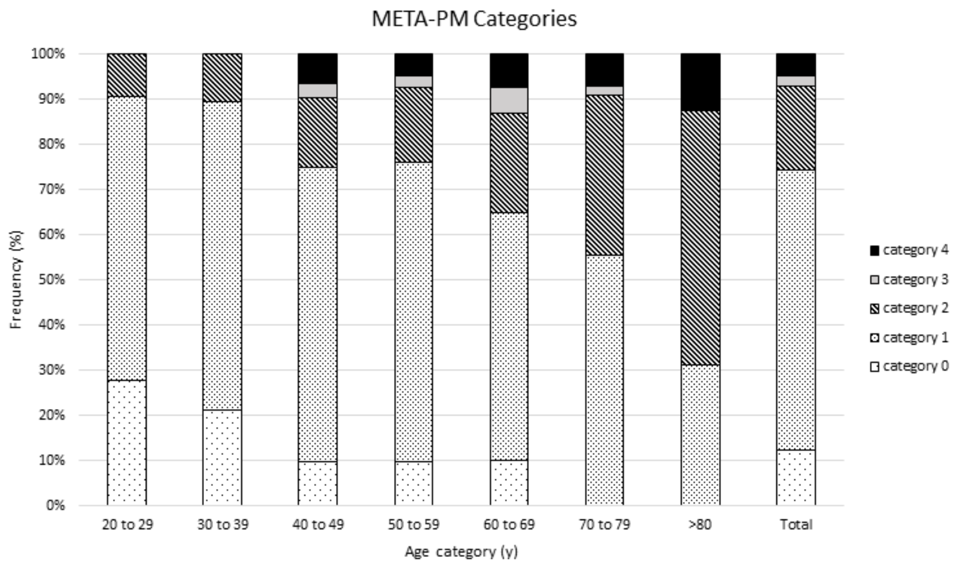

eFigure 6. Frequency of all META-PM categories in different age categories.

Staging of pathology was in accordance with META-PM: category 0 was defined as absence of any retinal complication; category 1 as presence of a tessellated fundus; category 2 as presence of diffuse hypopigmentation, i.e. diffuse chorioretinal atrophy; category 3 as presence of one or more patches of myopic atrophy not located in the central circle; and category 4 as presence of myopic atrophy located in the central circle.

© 2021 Haarman AEG et al. *JAMA Ophthalmology.*

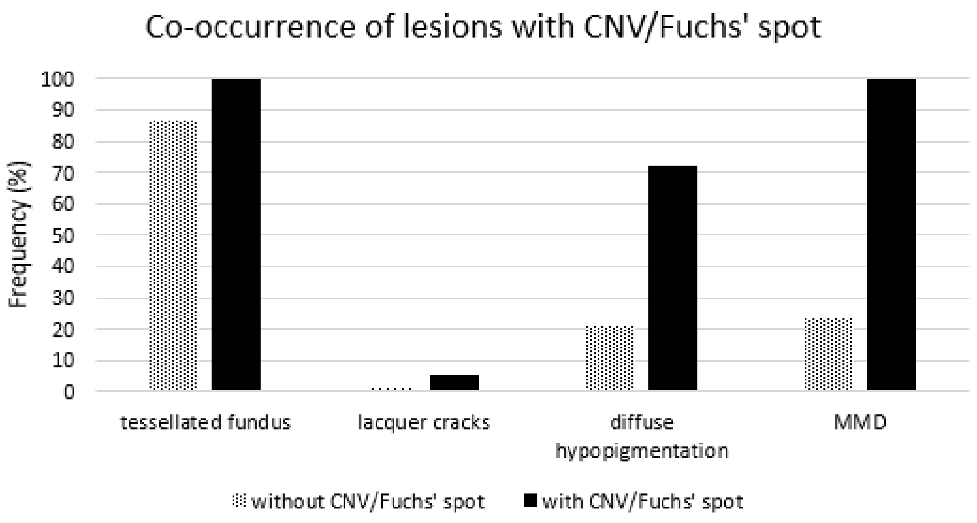

***eFigure 7. Frequency of tessellated fundus, lacquer cracks, diffuse hypopigmentation and MMD in eyes with and without CNV or Fuchs' spot**. Abbreviations: CNV=choroidal neovascularization.*

© 2021 Haarman AEG et al. *JAMA Ophthalmology.*

eTable 1. Association between various myopic features and axial length, spherical equivalent and age.

Variable	Myopic feature	Adjusted OR[a]	95%CI
**AL (mm)**	Tessellated fundus	2.030	1.588-2.594
	MMD	2.553	2.133-3.056
	Staphyloma	2.514	2.104-3.005
	Diffuse hypopigmentation	2.154	1.837-2.525
	RPE hyperpigmentation	2.563	2.074-3.166
	CNV/Fuchs' spot	1.650	1.309-2.079
	Lacquer Cracks	1.583	1.232-2.034
	PPA	1.941	1.641-2.297
	PICC	1.250	0.977-1.600
	Tilted Disc	1.531	1.329-1.763
**SER (D)**	**Myopic feature**	**Adjusted OR[a]**	**95%CI**
	Tessellated fundus	0.793	0.705-0.892
	MMD	0.700	0.649-0.755
	Staphyloma	0.714	0.664-0.768
	Diffuse hypopigmentation	0.747	0.698-0.800
	RPE hyperpigmentation	0.685	0.628-0.748
	CNV/Fuchs' spot	0.826	0.736-0.926
	Lacquer Cracks	0.762	0.660-0.880
	PPA	0.822	0.765-0.883
	PICC	0.885	0.779-1.005
	Tilted Disc	0.835	0.776-0.897
**Age (y)**	**Myopic feature**	**Adjusted OR[b]**	**95%CI**
	Tessellated fundus	1.050	1.030-1.070
	MMD	1.061	1.043-1.079
	Staphyloma	1.047	1.030-1.064
	Diffuse hypopigmentation	1.056	1.039-1.074
	RPE hyperpigmentation	1.077	1.049-1.105
	CNV/Fuchs' spot	1.082	1.040-1.125
	Lacquer Cracks	1.003	0.953-1.054
	PPA	1.037	1.024-1.051
	PICC	1.005	0.967-1.044
	Tilted Disc	1.015	0.994-1.036

[a] Adjusted for age and sex. [b] Adjusted for SER and sex. Abbreviations: AL=axial length; D=diopters; SER= spherical equivalent; y=years; RPE=retinal pigment epithelium; CNV= choroidal neovascularization; PPA= peripapillary atrophy; PICC: peripapillary intrachoroidal cavitation.

© 2021 Haarman AEG et al. *JAMA Ophthalmology.*

eTable 2. Frequency of Myopic Macular Degeneration (MMD) and Meta-PM Categories 2-4 in the Rotterdam Study.

Factor	Total	MMD		Meta-PM category 2 Diffuse chorioretinal atrophy		Meta-PM category 3 Patchy chorioretinal atrophy		Meta-PM category 4 Myopic macular atrophy	
	N	*N=40*		*N=34*		*N=1*		*N=5*	
		n	%	n	%	n	%	n	%
**Total**	117	40	34.2%	34	29.1	1	0.9	5	4.3
**Age**	Mean (95%CI)	40	75.3 (72.6-77.9)	34	75.5 (73.0-78.1)	1	69.7	5	74.8 (56.1-93.4)
**Age group, y**									
**40 to 49**	4	0	0	0	0	0	0	0	0
**50 to 59**	21	1	4.8	0	0	0	0	1	4.8
**60 to 69**	36	11	30.6	9	25	1	2.8	1	2.8
**70 to 79**	40	17	42.5%	16	40	0	0	2	2.5
**>80**	16	11	68.8%	9	56.3	0	0	2	12.5
**P-value for trend**			<.001		.001		.69		.48
**Male**	48	15	31.3%	12	25	0	0	3	6.3
**Female**	69	25	36.2%	22	31.9	1	1.4	2	2.9
**Comparison sex[a]**			.58		.42		1		.40
**SER (D) (available N=117)**	Mean (95%CI)	40	-11.8 (-13.2to -10.4)	34	-11.3 (-12.7 to -9.9)	1	-18		-13.6 (-20.3 to-6.9)
**OR (95%CI) [b]**			0.577 (0.453-736)		0.758 (0.659-0.871)		NA		0.758 (0.612-0.940)
**AL (mm) (available N=65)**	Mean (95%CI)	17	28.1 (27.2-29.1)	14	28.2 (27.1-29.2)	NA	NA	3	28.1 (21.2-35.0)
**OR (95%CI) [b]**		5.484 (2.058-14.613)		3.352 (1.700-6.609)		NA		1.687 (0.891-3.193)	
**BCVA (Snellen)**	109	36	0.35 (0.26-0.44)	30	0.42 (0.33-0.50)	1	0.01	5	0.05 (0.0-0.15))
**Approximate Snellen equivalent at 20 feet**			20/57		20/48		20/2000		20/400
**Comparison BCVA[c]**				ref		NA		<0.001	

Frequencies are stratified by age, gender. SER, axial length and BCVA are also shown. MMD according to the META-PM classification system was defined as META-PM category ≥2 or presence of any 'plus' lesions (i.e. choroidal neovascularization (CNV), Fuchs Spot, lacquer cracks). Sex was determined by the investigator. [a] P-value Chi-square test. [b]Adjusted for age and sex. [c] P-value independent T-test. Abbreviations: SER = spherical equivalent; D=diopters; AL = axial length; BCVA: best-corrected visual acuity

© 2021 Haarman AEG et al. *JAMA Ophthalmology.*

eTable 3. Frequency of Myopic Macular Degeneration (MMD) and Meta-PM Categories 2-4 in the Myopia Study (MYST).

Factor	Total	MMD		Meta-PM category 2 Diffuse chorioretinal atrophy		Meta-PM category 3 Patchy chorioretinal atrophy		Meta-PM category 4 Myopic macular atrophy	
	N	*N=122*		*N=81*		*N=14*		*N=25*	
		n	%	n	%	n	%	n	%
**Total**	509	122	24.0	81	15.9	14	2.8	25	4.9
**Age**	Mean (95%CI)	122	53.1 (51.0 – 55.2)	81	50.9 (48.2-53.5)	14	57.4 (51.9-62.9)	25	56.7 (52.4-61.0)
**Age group, y**									
**20 to 29**	65	6	9.2	6	9.2	0	0	0	0
**30 to 39**	94	10	10.6	10	10.6	0	0	0	0
**40 to 49**	120	31	25.8	19	15.8	4	3.3	8	6.7
**50 to 59**	142	39	27.5	27	19.0	4	2.8	7	4.9
**60 to 69**	72	27	37.5	15	20.8	5	6.9	7	9.7
**70 to 79**	16	9	56.3	6	25	1	6.3	3	18.8
**P-value for trend**			<.001		.007		.005		<.001
**Male**	191	47	24.6	28	14.7	5	2.6	13	6.8
**Female**	318	75	23.6	53	16.7	9	2.8	12	3.8
**Comparison sex** [a]			.80		.55		.89		.13
**SER (D) (available for N=436)**	Mean (95%CI)	120	-13.1 (-13.9 to -12.2)	70	-12.6 (-13.4 to -11.7)	10	-14.0 (-17.2 to -10.7)	19	-14.6 (-16.9 to -12.2)
**OR (95%CI)** [b]		0.726	(0.669-0.788)	0.821	0.765-0.882	0.808	0.706-0.926	0.761	0.680-0.851
**AL (mm) (available for N=507)**	Mean (95%CI)	120	29.3 (28.9-29.7)	80	28.8 (28.4- 29.2)	13	30.0 (28.2-31.0)	25	30.8 (30.0-31.8)
**OR (95%CI)** [b]		2.451	2.034-2.953	1.520	1.328-1.741	1.509	1.197-1.902	2.079	1.655-2.611
**BCVA (Snellen)**	Mean (95%CI)	122/509	0.66 (0.59-0.73)	81/509	0.80 (0.74-0.87)	14/509	0.59 (0.37-0.82)	25/509	0.27 (0.15-0.39)
**Approximate Snellen equivalent at 20 feet**			20/30		20/25		20/34		20/74
**Comparison BCVA**[c]					ref		0.023		<0.001

Frequencies are stratified by age, gender. SER, axial length and BCVA are also shown. MMD according to the META-PM classification system was defined as META-PM category ≥2 or presence of any 'plus' lesions (i.e. choroidal neovascularization (CNV), Fuchs Spot, lacquer cracks). Sex was determined by the investigator. [a] P-value Chi-square test. [b] Adjusted for age and sex. [c] P-value independent T-test. Abbreviations: SER = spherical equivalent; D=diopters; AL = axial length; BCVA: best-corrected visual acuity.

© 2021 Haarman AEG et al. *JAMA Ophthalmology.*

eTable 4. Prevalence of various myopic complications concerning populations with Asian ethnicity.

Study	Chen et al.	Koh et al.	Chang et al.	Liu et al.	Xiao et al.	Zhao et al.	Chen et al.	Wong et al.	Wong et al.	Wong et al.
**Year of publication**	2012	2013	2013	2018	2018	2020	2012	2021	2021	20 21
**Total study population (N)**	337	593	332	857	884	1841 eyes	44	818	252	309
**Study type**	cross sectional, retrospective	Cross sectional, case-control	cross sectional	cross sectional	cross sectional	cross sectional case series	cross-sectional	cross-sectional	cross-sectional	cross-sectional
**Part of cohort study**	No	No	SP2, SiMES, SINDI	ZOC-BHVI	ZOC-BHVI	No	The Shihpai Eye Study	Shanghai High Myopia Study	Kangbuk Samsung Hospital Study	Hong Kong High Myopia Study
**Age (y)**	40,6 (17.1)	21,1 (1.2)	>39 y, 43.9% was >=60 years	22,2 (12.1)	7-70 y (median 19)	32.6 (18.10) in C0 group	≥65	30-90	15-90	30-79
**Sex (N Male (%))**	139 (41)	593 (100)	147 (44.3)	402 (46.9)	410 (64.4)	624 (33.9)	23	NA	NA	NA
**Ethnicity**	Chinese	Singapore	Singapore (Chinese, Malay, Indian)	Chinese	Chinese	Chinese	Chinese (Taiwan)	Chinese	South-Korea	Hong-Kong
**SER (D)**	-11.40 (4.80)	-8.87 (2.11)	18% <-10 D	-9.9 (3.4)	<-6	<-6	<-6D	<-5D	<-5D	<-5D
**AL (mm)**	-	27.45 (1.17)	24.5% > 27.68	27.4 (1.5)	>26	≥26.5	NA	29.6 (2.3)	28.0 (1.9)	27.4 (1.2)
**Grading method**	Avila et al. (m0-M5) and META-PM	META-PM	Avila et al. (m0-M5)	META-PM	META-PM	META-PM	Avilla et al.	META-PM	META-PM	META-PM
**Definition MMD**	>=C2 or plus lesion	>=C2	>=M2	>=C2	>=C2 or plus lesion	>=C2	≥M3	>=C2 or plus lesion	>=C2 or plus lesion	>=C2 or plus lesion
**Source of study population**	hospital based	population based, pre-employment screening, refractive error–stratified random sampling strategy	high myopia population from 3 population-based surveys	hospital based	hospital based	hospital based	population based	hospital based	hospital based	population based

© 2021 Haarman AEG et al. *JAMA Ophthalmology.*

**Tessellated fundus C1**	56 (9.3)	544 (91.7) [a]	196 (59.4)	NA	177 (20)	779 (42.3)	5 (11.4)	553 (67.6) [a]	201 (79.7) [a]	284 (91.9) [a]
**Diffuse chorioretinal atrophy C2**	19 (4.6)	31 (5.2)	64 (19.3)	177 (20.6)	178 (20.2)	524 (28.5)	8 (18.2)	101(12.3)	31 (12.3)	24 (7.8)
**Patchy chorioretinal atrophy C3**	35 (5.8)	11 (1.9)	18 (5.5)	25/890(2.8)	23 (2.6)	352 (19.1)	8 (18.2)	138 (16.9)	12 (4.8)	1 (0.3)
**Macular chorioretinal atrophy C4**	23 (3.8)	7 (1.2)	18 (5.5)	21/890 (2.4)	2 (0.2)	128 (7.0)	17 (38.6)	26 (3.2)	8 (3.2)	0 (0.0)
**CNV**	84 (20.7)	2 (0.34)	3 (0.9)	2/890 (0.22)	2 (0.2)	65 (3.53)	NA	64 (7.8) [b]	12 (4.8) [b]	NA
**Fuchs Spot**	NA	2 (0.34)	0 (0)	NA	2 (0.2)	183 (9.94)	NA	64 (7.8) [b]	12 (4.8) [b]	NA
**Lacquer Cracks**	176 (29.1)	6 (1)	6 (1.8)	16/857 (1.9)	17 (1.9)	151 (8.2)	7 (15.9)	30 (3.7)	18 (7.1)	NA
**MMD**	387 (64)	49 (8.3)	101 (30.5)	223 (25.8)	224 (25.3)	1004 (54.6)	32 (72.7)	265 (32.4)	51 (20.2)	25 (8.1)

Data are shown as mean (SD) for continuous variables and number (%) for categorical variables. C1-4 indicates META-PM category 1-4. [a] Includes META-PM C0 + C1. [b] Fuchs'Spot and CNV. Abbreviations: NA= not available; y=years; SER = spherical equivalent; AL=axial length; D= diopters; SP2 = Singapore Prospective Study Program; SiMES = the Singapore Malay Eye Study; SINDI = the Singapore Indian Eye Study; ZOC-BHVI = Zhongshan Ophthalmic Center-Brien Holden Vision Institute high myopia cohort study.

© 2021 Haarman AEG et al. *JAMA Ophthalmology.*
